# Self-concept 6 months after traumatic brain injury and its relationship with emotional functioning

**DOI:** 10.3389/fpsyg.2022.995436

**Published:** 2022-11-03

**Authors:** Guido Mascialino, Viviana Cañadas, Jorge Valdiviezo-Oña, Alberto Rodríguez-Lorenzana, Juan Carlos Arango-Lasprilla, Clara Paz

**Affiliations:** ^1^Escuela de Psicología y Educación, Universidad de Las Américas, Quito, Ecuador; ^2^Department of Psychology. Virginia Commonwealth University, Richmond, VA, United States

**Keywords:** traumatic brain injury, repertory grid, personal construct theory, self-concept, emotional functioning

## Abstract

This is an observational exploratory study assessing self-concept and its association with depression, anxiety, satisfaction with life, and quality of life 6 months after experiencing a traumatic brain injury. Participants were 33 patients who suffered a traumatic brain injury 6 months before the assessment. The measures used in this study were the Repertory Grid Technique, Patient Health Questionnaire-9, Generalized Anxiety Disorder-7, Satisfaction With Life Scale, and the Quality of Life after Brain Injury. We calculated Euclidean distances to assess differences in pre-and post-injury self-perception, as well as the proportion of opposed pole construct rating and polarization to understand how they are associated with the scores of the other offered measures. We found that the distance between the present and ideal self, as well as the distance between the present self and the self before the lesion showed moderate positive correlations with depression, and negative correlations with satisfaction with life and quality of life. Also, for the present and self before the lesion, the proportion of opposed pole ratings was correlated with depression symptoms, quality, and satisfaction with life, while for the present self and the ideal self this proportion was correlated with all the measures. The proportion of polarization of the present self and the total polarization was negatively correlated with symptom measures. The repertory grid might facilitate a greater understanding of self-concept after traumatic brain injury. This information could be used to guide treatments that address the emotions related to distances observed in the perception of the self.

## Introduction

Self-concept refers broadly to the internal representation of feelings and thoughts that a person has about themselves (Campbell et al., 1996). Self-concept is continuously shaped through the interaction between internal factors, such as biological and cognitive, and external factors such as the social environment, culture, and life events (Jetten et al., 2011). As a result, individuals construct their sense of self through the interplay between their identity, which identifies them as unique and different from others, and social identities based on their perceived group memberships (Tajfel and Turner, 1986). Research shows that self-concept is quite stable throughout a person’s life (Church et al., 2012); however, this stability might be disrupted by significant life events (Markus and Kunda, 1986) such as neurological injuries, major life transitions, social evaluation, and interventions targeting self-schema (Beadle et al., 2016).

A traumatic brain injury (TBI), formally defined “as an alteration in brain function, or other evidence of brain pathology, caused by an external force,” Menon et al. (2010, 1638) is a major event that might significantly destabilize a person’s life. TBI is a worldwide critical health issue with an annual incidence of 69 million persons globally, and a growing number of cases every year (Dewan et al., 2019). For those who survive, recovery depends on several factors, most importantly the severity of the injury, and can take from weeks to years. A 10-year retrospective study showed that, for the majority of cases, attaining a significant recovery could take up to 6 years (Huang et al., 2010).

A person’s life after TBI presents with many challenges stemming from physical, cognitive, behavioral, and emotional changes post-injury (Corrigan and Hammond, 2013). The associated deficits that result from this kind of injury, affecting cognitive (e.g., poor concentration, memory difficulties), physical (e.g., headache, dizziness), emotional (e.g., irritability, depression) or motor skills, often negatively impact the everyday functioning of the individual (Lundin et al., 2006). Consequently, it is common to experience psychological distress over time (Pagulayan et al., 2008).

The majority of people with TBI report symptoms of depression at least once, and significantly more times than people that have not suffered this kind of injury (Jorge et al., 2004; Hart et al., 2012). Furthermore, people with TBI and major depressive disorder are more susceptible to present comorbid anxiety (Jorge et al., 2004; Bombardier et al., 2010) as well as a lower quality of life (Bombardier et al., 2010). Moreover, people with TBI are prone to experience aggressive behaviors, suicidal ideation (Tsaousides et al., 2011; Mackelprang et al., 2014), and lower life satisfaction (Juengst et al., 2015). Social support is also affected. Persons with TBI report poor satisfaction with social support and lower locus of control in comparison with persons without TBI (Izaute et al., 2008). Lastly, emotional distress in persons with TBI has been associated with changes in self-concept (Beadle et al., 2016).

Changes in self-concept in this population have been studied using instruments that try to capture pre-post injury characteristics regarding self-concept, self-esteem, trauma, and personality (Tyerman and Humphrey, 1984; Nochi, 1998; Cantor et al., 2005; Rush et al., 2006; Cloute et al., 2008; Gracey et al., 2008; Muenchberger et al., 2008; Carroll and Coetzer, 2011; Gelech and Desjardins, 2011; Jones et al., 2011; Douglas, 2013; Downing et al., 2013; Lapadatu, 2015; Reddy et al., 2015; Riley and Hagger, 2015). All these studies show that pre-injury self-concept is more positive than post-injury self-concept. Overall, patients with TBI reported significant self-concept change, psychological distress (anxiety and depression) related to the change, strong correlations between affective distress and self-discrepancies, sexual changes related to fatigue, low confidence, pain, decreased mobility, feelings of being unattractive, construction of TBI as a deficit, loss of self in terms of unclear self-knowledge about their characteristics and capabilities and feelings of shame about the injury, among others (Tyerman and Humphrey, 1984; Nochi, 1998; Cantor et al., 2005; Rush et al., 2006; Cloute et al., 2008; Gracey et al., 2008; Muenchberger et al., 2008; Carroll and Coetzer, 2011; Gelech and Desjardins, 2011; Douglas, 2013; Downing et al., 2013; Reddy et al., 2015). Additionally, some studies indicate that people who have acquired a TBI have shown the need to grieve their previous selves in order to reconstruct their current selves (Nochi, 1998; Gelech and Desjardins, 2011). Cognitive inflexibility has also been proposed as a mechanism that sustains psychological symptoms after a traumatic brain injury, including poor adjustment to changes post-injury (Faulkner et al., 2020). Inflexibility could affect a person’s response to their injury and lead to poor outcomes (Broshek et al., 2015).

Changes in self-concept have been studied using several different methods, one of which is the repertory grid technique (RGT). This method was developed by Kelly (1955) as a tool to understand the personal constructs that every individual uses, and it was originally known as *The Rep Test* (Feixas and Cornejo, 1996; Fransella et al., 2004). For Kelly, personal constructs are the basic units of knowledge and analysis —not isolated but interconnected in a hierarchical network— which allow the individual to make predictions about themselves and their surroundings. Personal constructs are bipolar discriminations that can be described through verbal tags, such as *happy-sad* or *captivating-boring*, and can also operate as pre-verbal constructions to make life experiences more predictable (Bannister, 2003). The RGT has several methodological variants in form and administration.

Gracey et al. (2008) used a variation of the RGT to explore the construction of meanings and self-concept of 32 individuals with a brain injury through a group exercise that allowed eliciting the constructs by comparing pre-injury, current and ideal selves. The elicited constructs were then classified using thematic analysis. Most of the constructs exhibited themes related to the individual’s experience of the self in the world and their functioning in social and practical activity. That is the only study that has employed the RGT with individuals who have acquired a TBI; however, it only considered it as a qualitative method while the repertory grid could also provide significant quantitative information. Moreover, Gracey et al. (2008) applied the technique in a group scenario, while to understand the personal meanings it will be useful to apply it in an individual scenario.

Research about self-concept after TBI is limited in comparison to research looking at changes in cognition and general functioning (Bryson-Campbell et al., 2013; Manley et al., 2017; McInnes et al., 2017; Tsai et al., 2021; Villa et al., 2021). The current study aims to contribute to this body of knowledge by exploring ways of self-construing post-injury. Specifically, this study will explore: (a) whether distances between pre- and post-injury self-concept relate to mental health, and (b) whether a polarized view of self relates to mental health. The RGT is particularly suited for this endeavor due to its theoretical and methodological underpinnings explained above. Our study aims to use the RGT as a source of quantitative information and present it as a tool to assess the distances between individuals’ views of their present, past, and ideal selves, as well as polarization of self-concept, and their possible relations with depression, anxiety, satisfaction with life, and quality of life 6 months after experiencing a TBI. Given ample evidence demonstrating the relationship between self-concept discrepancy, polarization, and psychological symptoms, we expect to find a similar relationship in this population (Feixas et al., 2008, 2021). Due to the novelty of the use of the RGT for this purpose and with people with a TBI, the present work is proposed as an exploratory study.

## Materials and methods

The Research Bioethics Committee of the Eugenio Espejo Hospital approved the protocol of the present study. This research study was performed in accordance with the principles stated in the Declaration of Helsinki. The data presented here were collected as part of a larger longitudinal study tracking recovery from TBI. The data for the larger longitudinal study were collected between January 2018 and March 2020 at three assessment points: hospitalization, six (±2) months post-injury, and 12 (±2) months after the injury. For this study, we report information from participants assessed six (±2) months post-injury.

### Participants

Before including the participants, we used the Galveston Orientation and Amnesia Test (GOAT; Levin et al., 1979) to estimate if the patients had recovered from post-traumatic amnesia. The test includes several orientation questions such as *What is your name?* and *What day of the week is it?* A score of 75 out of 100 is considered evidence of emergence from post-traumatic amnesia. Those who scored 75 and above 2 days in a row were included in the study. Thirty-three participants who suffered a TBI were included in this study and completed an assessment six (±2) months after the first assessment conducted when they were hospitalized, 31 (93.94%) were men, and 2 were women (6.06%). Patients were hospitalized for periods ranging from 2 days to 47 days, with a mean of 13.30 days and a median of 7 days. Their ages ranged from 18 to 61 years (*M* = 36 [31.90, 40.15], SD = 12.69). Regarding work status prior to the injury, 17 (52%) participants reported working full-time, seven (21%) working part-time, four (12%) were students who did not work, two (6%) were unemployed, and three (9%) persons did not respond. In total, 19 (58%) had a mild TBI, 10 (30%) a severe one, and 4 (12%) a moderate one.

### Instruments

#### Repertory grid technique

The RGT is a tool whose goal is to capture personal construct systems. It was designed as a semi-structured interview in which the individual considers a sample of elements of their world, such as aspects of the self and significant others (e.g., present self and ideal self, parents, siblings, couple, friends) at different time points. The interviewees are asked to search for resemblances and differences between them by eliciting bipolar constructs such as *captivating-boring,* on a 7-point Likert scale. Once the constructs are elicited in this manner, the individual rates each element according to each construct. The result is a matrix of numbers that can be analyzed using quantitative methods to attain several measures (Bannister, 2003). In this study, the elements included were how I am (present self); how I was before the injury (self before the lesion); and how I would like to be (ideal self).

#### Patient health questionnaire-9

The Patient Health Questionnaire-9 (PHQ-9) is a self-report measure created to assess depression based on its criteria according to the DSM-IV (Kroenke et al., 2001). It has nine items —such as *Feeling down, depressed, or hopeless* and *Thoughts that you would be better off dead or of hurting yourself in some way*— whose possible answers range from “0” (not at all) to “3” (nearly every day). It has shown high sensitivity and specificity, as well as reliability and validity to assess depression severity (Kroenke et al., 2001; Martin et al., 2006; Cameron et al., 2008; Hinz et al., 2016; Muñoz-Navarro et al., 2017; Urtasun et al., 2019). Scores of five, 10, 15, and 20 correspond to mild, moderate, moderately severe, and severe depression, respectively (Kroenke et al., 2001). Several studies have shown its reliability, validity, and clinical utility to assess depression following a TBI (Fann et al., 2005; Donders and Darland, 2017; Donders and Pendery, 2017; Teymoori et al., 2020). The reliability of this measure in this study was acceptable (*α* = 0.79).

#### Generalized anxiety disorder-7

The Generalized Anxiety Disorder-7 (GAD-7) is a brief seven-item self-administered measure designed to assess generalized anxiety disorder (GAD; Spitzer et al., 2006). The participants answer items such as *Feeling nervous, anxious or on edge* and *Not being able to stop or control worrying*. Its possible answers range from “0” (not at all) to “3” (nearly every day). It has shown favorable internal consistency, as well as criterion, construct, factorial, and procedural validity. It has also shown adequate sensitivity and specificity. The GAD-7 is a useful instrument for detecting GAD and evaluating its severity (Spitzer et al., 2006; Johnson et al., 2019). Its internal consistency, validity, and clinical utility to screen and identify anxiety disorders after TBI has been reported (Teymoori et al., 2020; Zachar-Tirado and Donders, 2021). The reliability of this measure in this study was acceptable (*α* = 0.71).

#### Satisfaction with life scale

The Satisfaction With Life Scale (SWLS) was developed to measure global life satisfaction (Diener et al., 1985). It is comprised of five items such as *In most ways my life is close to my ideal* and *I am satisfied with my life*. Each item is scored on a one to seven Likert scale, so the total score ranges from “5” (extreme dissatisfaction) to “35” (extreme satisfaction). The SWLS has shown high reliability and validity (Diener et al., 1985; Pavot and Diener, 2008). Scores between 5 and 9 show the participant is extremely dissatisfied with life, between 10 and 14 dissatisfied, between 15 and 19 slightly dissatisfied, a score of 20 representing the neutral point on the scale, between 21 and 25 show the participant is slightly satisfied, between 25 and 29 a high satisfaction level, and scores between 31 and 35 suggest the participant is extremely satisfied with life (Pavot and Diener, 2008). Its psychometric properties for patients who have suffered a TBI have been explored and it has been shown to be a valuable measure to monitor satisfaction with life following a TBI (Corrigan et al., 2001; Amtmann et al., 2019). The reliability of this measure in this study was acceptable (*α* = 0.77).

#### Quality of life after brain injury

The Quality of Life after Brain Injury (QOLIBRI) is a disease-specific measure comprised of 37 items that assesses health-related quality of life (HRQoL) regarding physical condition, thinking activities, feelings and emotions, day-to-day functioning, relationships and social/leisure activities, present condition, and future expectations (Von Steinbuechel et al., 2005). The QOLIBRI includes questions such as *How satisfied are you with your ability to concentrate, for example when reading or keeping track of a conversation?* and *How satisfied are you with the extent of your independence from others?*

The QOLIBRI total scores are obtained by summing the scores on the 37 items (graded 1–5), so the maximum score is 185. Subsequently, that score is transformed to a 0–100 scale, where “0” represents the worst possible quality of life “100” represents the best possible quality of life. It has shown adequate psychometric properties and has shown its utility and sensitivity for patients from heterogeneous populations and cultural backgrounds (Von Steinbuechel et al., 2005; Truelle et al., 2010). The reliability of this measure in this study was excellent (*α* = 0.94).

### Procedure

Patients were first approached at the Eugenio Espejo Hospital, which is a tertiary hospital with 20 medical specialties and 15 surgical specialties. Before including any patient, they were evaluated with the GOAT to estimate if they had recovered from post-traumatic amnesia, i.e., if they scored 75 or higher on the GOAT 2 days in a row. Patients that complied with this criterion were invited to be part of the study; they received information regarding its aims, methods, risks, benefits and confidentiality. A researcher was available to participants in case they needed clarification with any questions about the research. All included participants or one of their relatives —if the patients were not capable— signed a written informed consent.

The patients’ contact information was collected in order to assess them after the hospitalization at follow-up six (±2) months after the injury. Consequently, 16 participants (48.48%) were assessed 6 months post-injury, 9 (27.27%) were assessed 5 months post-injury, 3 (9.09%) were assessed 7 months post-injury, 4 (12.12%) were assessed 8 months post-injury and 1 (3.03%) was assessed 4 months post-injury. They were evaluated on average 6 (SD = 1) months post-injury.

All measures, including the RGT were completed in paper format. Regarding the RGT, the constructs, presented as bipolar dimensions, were elicited as follows. First, the participants were asked to describe themselves in the present and before the injury. Second, the interviewer asked about similarities and differences between their present and past self-perception. The descriptions resulted in adjectives that represented one pole of the construct. Then, the participants were asked to indicate the opposite of the first adjective to determine the other pole. Third, once the participants were unable to elicit more constructs, the interviewer presented a list of adjectives extracted from the semantic differential scale created by Tyerman and Humphrey (1984). This list presents 40 adjectives that are relevant to people with head injuries. Each adjective was read to the participants, and they picked those that best fitted their description of present, past and ideal self. To ensure that all pairs made sense, the participants were asked to review them. Fourth, the participants were asked to score each construct according to each element using a seven-point Likert scale where one, two, and three indicate different levels of the left pole of the construct; four represents the middle point; and five, six, and seven are different levels of the right pole. The interview ended when all the elements were assessed according to all the constructs.

### Data analysis

#### Descriptive analysis

We conducted descriptive analyses of sociodemographic variables and measures. For the participants, we calculated frequencies regarding the total number, and for the measures, we calculated frequencies based on categorical levels. Furthermore, we calculated means with 95% bootstrapped confidence intervals (CIs) for the participants’ ages and the questionnaires’ total scores using the R’s CECPfuns package (Evans, 2021), as well as standard deviations using R’s stats package (R Core Team, 2021).

#### RGT indexes

Several indexes from the RGT were extracted as follows:

##### Euclidean distances

We calculated the standardized Euclidean distances (ED) as proposed in Salla et al. (2015). This analysis allows determining the distance between two elements (e.g., present-self and ideal self) expressed as a decimal number between zero and one, where one represents the maximum distance. The standardized ED is calculated through Equation 1:


(1) $${\mathrm{ED}}_{\left(x,y\right)}=\left\{{\left[\underset{i}{\sum }{\left(x_{i}-y_{i}\right)}^{2}\right]}^{1/2}/\left[\mathrm{MD}\left(C^{1/2}\right)\right]\right\}$$

where *x* and *y* are the elements to be compared, MD is the maximum difference between scores, and *C* is the total number of constructs in the grid. The EDs were calculated for all the possible combinations of pairs of elements within each grid, in total six pairs. Furthermore, mean EDs were calculated for all combinations of pairs.

##### Proportion of opposed pole construct rating

To understand the magnitude of the distance between the pairs of elements, we considered that it would be useful to identify the proportion of constructs by pairs of elements in which the participants rated the construct at one pole on one element (rating one, two or three) and at the opposite pole on the other element of the pair (five, six or seven). For example, for the construct *happy-sad,* there was opposed construct rating if a participant rated the present self as *Very sad* (7), and the self before the lesion as *Somewhat happy* (3).

##### Proportion of polarization

Since our purpose was to understand the different perceptions of the self, we calculated the number of constructs that were scored one or seven for each element and in total in the whole matrix, denoting extreme perceptions of the self. The proportion of polarization of the whole matrix has been previously used as a measure of cognitive rigidity in women with fibromyalgia (Aguilera et al., 2019).

#### Correlations with bootstrapped 95% CIs

We calculated Spearman correlations with non-parametric bootstrapped 95% CIs: (1) between the self-reported measures (PHQ-9, GAD-7, SWLS. QOLIBRI) scores 6 months after the injury, (2) between Euclidean distances and self-reported measures scores. (3) between polarization and the self-reported measures scores, and (4) between opposed construct rating by pair of elements and the self-reported measures scores, using the R’s CECPfuns package (Evans, 2021). Furthermore, we calculated the *p*-values for these correlations with R’s stats package (R Core Team, 2021).

## Results

Six months after the injury, the mean depression score was 7.24 [5.42, 9.12] (SD = 5.40). Twenty-three (70%) patients presented mild depression and 10 (30%) presented moderate-to-severe depression. The mean anxiety score was 5.54 [4.12, 6.96] (SD = 4.14) Twenty-seven (82%) patients showed mild anxiety and six (18%) moderate-to-severe anxiety.

Regarding satisfaction with life 6 months after the injury, six (18%) participants were dissatisfied, four (12%) were slightly dissatisfied, four (12%) were slightly satisfied, 15 (45%) showed high satisfaction and four (12%) patients were extremely satisfied. Mean satisfaction with life was 23.30 [20.72, 25.54] (SD = 7.17).

Concerning quality of life 6 months after the injury, considering the proposed cutoff (<60) for identifying low or impaired HRQoL with the QOLIBRI (Truelle et al., 2010; Wilson et al., 2017), 12 (36%) participants showed low or impaired HRQoL. The mean quality of life score was 61.19 [54.87, 66.97] (SD = 16.39).

The mean number of constructs elicited in the RGT was 19 (SD = 3.90) in total, 8 (SD = 3.57) self-generated, and 11 (SD = 4.31) elicited from the list. The Euclidean distance between present-self and self-before the lesion was 0.39 [0.31, 0.47], between present-self and ideal-self was 0.35 [0.29, 0.43] and between the self before the lesion and the ideal self was 0.37 [0.31, 0.43]. As the CIs of all these distances overlap, there is no evidence of significant differences between them.

Table 1 shows the correlations between all the self-reported measures. The PHQ-9 shows a significant moderate negative correlation with the SWLS and the QOLIBRI, and a significant high positive correlation with the GAD-7. That is, the higher the depression score is, the lower the satisfaction with life and quality of life scores, and the higher the depression score is, the higher is the score on the anxiety measure. We also found significant correlations of the GAD-7 with the SWLS as well as with the QOLIBRI, both low and negative.

**Table 1:** Spearman’s correlations between questionnaire scores 6 months after the injury.

Measure	PHQ-9	GAD-7	SWLS
	Spearman’s rho [95% CI]^a^	Spearman’s rho [95% CI]^a^	Spearman’s rho [95% CI]^a^
QOLIBRI	−0.67 [−0.85, −0.40]***	−0.48 [−0.72, −0.15]**	0.76 [0.58, 0.86]***
SWLS	−0.53 [−0.73, −0.20]**	−0.37 [−0.69, −0.01]*	
GAD-7	0.72 [0.47, 0.87]***		

* indicates *p* < 0.05; ** indicates *p* < 0.01; *** indicates *p* < 0.001.

a 95% bootstrapped confidence intervals.

Table 2 shows correlations for each score of the self-reported measures against the Euclidean distances between all the possible pairs of elements. The distance between present self and the self before the lesion showed significant correlations with the scores on all measures except the GAD-7 scores: a low positive correlation with the PHQ-9, and low negative correlations with the SWLS, and the QOLIBRI. The distance between the present self and the ideal self also showed significant correlations with the scores on all measures except the GAD-7 scores: a low positive correlation with the PHQ-9 score, a low negative correlation with the SWLS and a moderate negative correlation with the QOLIBRI.

**Table 2:** Spearman’s correlations between Euclidean distances with the scores of self-reported measures.

	PHQ-9	GAD-7	SWLS	QOLIBRI
	Spearman’s rho [95% CI]^a^	Spearman’s rho [95% CI]^a^	Spearman’s rho [95% CI]^a^	Spearman’s rho [95% CI]^a^
Present self – self before the lesion	0.35 [0.01, 0.63]*	0.25 [−0.08, 0.52]	−0.37 [−0.63, −0.03]*	−0.49 [−0.74, −0.16]**
Present self – ideal self	0.46 [0.18, 0.70]*	0.34 [−0.05, 0.67]	−0.42 [−0.71, −0.04]*	−0.61 [−0.82, −0.32]***
Self before the lesion – ideal self	0.08 [−30, 0.45]	0.01 [−0.37, 0.42]	−0.28 [−0.59, 0.06]	−0.12 [−0.49, 0.29]

* indicates *p* < 0.05; ** indicates *p* < 0.01; *** indicates *p* < 0.001.

a 95% bootstrapped confidence intervals.

Also, we calculated the proportion of opposed pole construct ratings by pairs of elements. Table 3 shows these proportions and their correlations with all the self-reported measures. For the pair *present self and self before the lesion* we found significant correlations with the PHQ-9 (low positive), and with the SWLS and the QOLIBRI (moderate negative). The pair *present self and ideal-self* showed significant correlations with all the self-reported measures: moderate positive with the PHQ-9, low positive with the GAD-7, moderate negative with the SWLS and high negative with the QOLIBRI. The pair self before the lesion and ideal self, showed a significant low negative correlation with the SWLS.

**Table 3:** Spearman’s correlations between proportion of opposed construct rating by pair of elements with scores of self-reported measures.

	*M* [95% CI]	PHQ-9	GAD-7	SWLS	QOLIBRI
		Spearman’s rho [95% CI]^a^	Spearman’s rho [95% CI]^a^	Spearman’s rho [95% CI]^a^	Spearman’s rho [95% CI]^a^
Present self – self before the lesion	0.38 [0.27, 0.49]	0.47 [0.14, 0.72]**	0.33 [0.04, 0.60]	−0.52 [−0.75, −0.18]**	−0.60 [−0.83, −0.31]***
Present self – ideal self	0.28 [0.19, 0.27]	0.55 [0.28, 0.74]***	0.46 [0.12, 0.72]**	−0.55 [−0.77, −0.24]***	−0.75 [−0.89, −0.52]***
Self before the lesion – ideal self	0.24 [0.17, 0.32]	0.12 [−25, 0.44]	0.09 [−0.27, 0.41]	−0.39 [−0.65, −0.03]*	−0.19 [−0.49, 0.22]

* indicates *p* < 0.05; ** indicates *p* < 0.01; *** indicates *p* < 0.001.

a 95% bootstrapped confidence intervals.

Finally, we calculated the proportion of polarization by element and for all the elements. The mean proportion for each element, the 95% CI and the correlations against all the self-reported measures scores are presented in Table 4. Significant correlations were found between present self-polarization and the PHQ-9 and GAD-7 scores, both correlations being low negative. Moreover, there was a low negative correlation between total polarization and the GAD-7 score.

**Table 4:** Spearman’s correlations between proportion of polarization and the self-reported measures scores.

	*M* [95% CI]^a^	PHQ-9	GAD-7	SWLS	QOLIBRI
		Spearman’s rho [95% CI]^a^	Spearman’s rho [95% CI]^a^	Spearman’s rho [95% CI]^a^	Spearman’s rho [95% CI]^a^
Present self	0.22 [0.16, 0.29]	−0.36 [−0.60, −0.01]*	−0.43 [−0.66, −0.12]*	0.22 [−0.12, 0.52]	0.17 [−0.20, 0.50]
Ideal self	0.55 [0.45, 0.64]	−0.06 [−0.39, 0.30]	−0.30 [−0.61, 0.08]	0.26 [−0.11, 0.57]	0.23 [−0.18, 0.55]
Self before the lesion	0.28 [0.21, 0.34]	−0.11 [−0.46, 0.27]	−0.16 [−0.50, 0.22]	−0.01 [−0.36, 0.31]	−0.11 [−0.43, 0.22]
Total polarization	0.35 [0.29, 0.41]	−0.19 [−0.53, 0.16]	−0.37[−0.63, −0.02]*	0.21 [−0.16, 0.54]	0.14 [−0.23, 0.47]

* indicates *p* < 0.05; ** indicates *p* < 0.01; *** indicates *p* < 0.001.

a 95% bootstrapped confidence intervals.

## Discussion

In this study, the RGT was used as a source of quantitative information to understand self-concept after a TBI and its possible relations with depression, anxiety, satisfaction with life, and quality of life measures. This adapted version of the technique seems adequate to obtain the necessary information to identify perceived distances between aspects in the personal construction of the self in this specific population.

In this study, 70% of the patients presented mild depression and 30% presented moderate-to-severe depression, 82% of the patients showed mild anxiety and 18% moderate-to-severe anxiety. Analysis of the element distance measure indicated no significant differences in how positively (in terms of proximity to the ideal self) patients view themselves after the lesion compared to how they have been before the lesion occurred. However, we found that the distance between present self and the ideal self showed a significant and positive association with the score representing symptoms of depression, and negative associations with the satisfaction with life and with the quality of life. Of note, a less polarized view of self-construing was associated with higher levels of depression and anxiety in the study participants. This result is not consistent with findings in other groups in which polarization and inflexibility are typically associated with lower levels of well-being (Feixas et al., 2008, 2021; Aguilera et al., 2019; Faulkner et al., 2020). It is possible that, 6 months post-injury, a less well-defined sense of self can result in greater distress.

Levels of congruence or incongruence in several kinds of self-representation have been linked to emotional problems such as depression, consistent with the results of the current study (Higgins et al., 1986; Higgins, 1987). Discrepancy in aspects of self-concept, such as differences between present- and ideal-self, has been well documented in other populations (Feixas et al., 2008, 2021). Consistent with this, our findings suggest that viewing the current self as dissimilar to both the pre-lesion self and the ideal self is associated with affective distress in people who have suffered a TBI and can be compared to those from Cantor et al. (2005) who examined the utility of self-discrepancy theory in relation to depression and anxiety post-TBI and found strong correlations between anxiety, depression, and self-discrepancies. However, we only found positive correlations with depression, and negative correlations with satisfaction with life and quality of life. These correlations between emotional distress and self-discrepancies may be related to people who have suffered a TBI perceiving their past and present selves in a pessimistic manner, as Shields et al. (2016) have pointed out.

Based on our results, the RGT can play an important role in TBI research, by exploring self-concept and correlates to emotional outcomes. However, it may also provide a platform to formulate treatment approaches. An increase in awareness of changes post-injury has been tied to better outcomes in neuropsychological treatments (Sherer et al., 1998; Hurst et al., 2018). Thus, clarifying the areas in which there is a distance between current and ideal self can itself be therapeutic by increasing awareness of deficits after TBI, thereby opening the door to address them in the neurorehabilitation process. Furthermore, exploring and resolving distances between pre- and post-injury self-concept could further a person’s adjustment to disability and improve mental health in the process. In addition, clarifying self-concept after injury may lead to a more well-defined sense of self and lessened psychological distress. Holistic approaches to neurorehabilitation indeed include both the recognition of changes in the pursuit of higher functioning as well as the construction of personal meaning within the new matrix of abilities, relationships, and expectations brought about by the traumatic brain injury (Ben-Yishay et al., 1985).

This study has limitations regarding sample size and gender representativeness. Further research using the RGT with a bigger sample of patients and more women participants that have suffered a TBI could lend further support to the notion that discrepancies and/or polarization of self-concept occur in this population and affect their mental health.

This is the first study to use quantitative analysis of the RGT to explore the relationship between aspects of self-construing and mental health in sufferers of traumatic brain injury, 6 months post-lesion. Mental health providers should consider exploring self-concept discrepancies and polarization when working with this population.

## Data availability statement

The raw data supporting the conclusions of this article will be made available by the authors, without undue reservation.

## Ethics statement

The studies involving human participants were reviewed and approved by Research Bioethics Committee of the Eugenio Espejo Hospital. The patients/participants provided their written informed consent to participate in this study.

## Author contributions

GM: study design, supervision, and paper writing. VC: paper writing. JV-O: data collection, analysis, and paper writing. AR-L: study design and paper writing. JA-L: paper writing. CP: study design and paper writing. All authors contributed to the article and approved the submitted version.

## Funding

This work was supported by the (Dirección General de Investigación y Vinculación, Universidad de Las Américas, Quito, Ecuador) under grant (PSI.GM.1705).

## Conflict of interest

The authors declare that the research was conducted in the absence of any commercial or financial relationships that could be construed as a potential conflict of interest.

## Publisher’s note

All claims expressed in this article are solely those of the authors and do not necessarily represent those of their affiliated organizations, or those of the publisher, the editors and the reviewers. Any product that may be evaluated in this article, or claim that may be made by its manufacturer, is not guaranteed or endorsed by the publisher.
